# Secondary Aortoduodenal Fistula Diagnosed after Repeated Gastrointestinal Bleeding Episodes in a Patient With Prior Aortic Graft Surgery

**DOI:** 10.1002/deo2.70181

**Published:** 2025-08-06

**Authors:** Jun Kubota, Chikamasa Ichita, Soichiro Nakaya, Takashi Nishino, Chihiro Sumida, Akiko Sasaki, Daisuke Hama

**Affiliations:** ^1^ Gastroenterology Medicine Center Shonan Kamakura General Hospital Kanagawa Japan; ^2^ Department of Cardiovascular Surgery Shonan Kamakura General Hospital Kanagawa Japan

**Keywords:** aortoduodenal fistula, contrast media allergy, diagnostic challenge, endovascular repair, gastrointestinal bleeding

## Abstract

Secondary aortoduodenal fistula (sADF) is a rare but life‐threatening complication after aortic graft surgery. Diagnosis is often challenging, particularly when contrast‐enhanced computed tomography (CT) and endoscopy results are inconclusive. We report a case in which diagnosis was challenging due to contrast media allergy and non‐diagnostic endoscopic findings, highlighting the importance of clinical suspicion in such scenarios. A 76‐year‐old man with a history of graft replacement for a ruptured abdominal aortic aneurysm presented with five episodes of gastrointestinal bleeding over 3 months. Despite multiple examinations, no bleeding source was identified. Eventually, after the patient developed hypovolemic shock, contrast‐enhanced CT was performed with informed consent, which revealed contrast extravasation from the aorta into the horizontal portion of the duodenum. Endoscopy subsequently identified an exposed vessel in the same location, confirming the diagnosis of sADF. Endovascular aortic repair was successfully performed. This case underscores the need to consider sADF in patients with aortic grafts who present with recurrent, unexplained gastrointestinal bleeding.

## 1 Introduction

Aortoduodenal fistula (ADF) is a rare but frequently fatal condition with mortality rates ranging from 28% to 44% [1, 2]. Specifically, secondary ADF (sADF), which develops after aortic graft surgery, is approximately 10 times more common than primary ADF and represents a critical complication in patients with a history of aortic intervention [1]. Although the classic triad of gastrointestinal bleeding, abdominal pain, and a pulsatile abdominal mass is traditionally described, it rarely presents in its entirety [3]. Instead, herald bleeding—recurrent episodes of minor gastrointestinal bleeding that precede massive hemorrhage—is frequently observed, and it serves as an important early diagnostic clue [2, 4].

Endoscopic examination alone may fail to detect conclusive indicators, making a combined assessment with computed tomography (CT) essential for diagnosis [2, 5]. Contrast‐enhanced CT may reveal extravasation of contrast medium from the aorta into the duodenum—a characteristic finding of ADF. However, this sign is often absent. Indirect CT findings, such as close anatomical proximity between the aorta and duodenum or the presence of air within the aortic lumen, may raise clinical suspicion [2].

We present a case of sADF in a patient who experienced recurrent gastrointestinal bleeding over 3 months. The diagnosis was significantly delayed because contrast‐enhanced CT, a key diagnostic modality, could not be performed initially due to contrast media allergy. Despite multiple endoscopic examinations and a non‐contrast CT, no definitive source of bleeding was identified. This case underscores the risk of delayed diagnosis when contrast‐enhanced CT cannot be performed and the critical need to consider sADF in patients presenting with recurrent unexplained gastrointestinal bleeding after aortic graft surgery.

## 2 Case

The patient was a 76‐year‐old man with a history of prosthetic graft replacement for a ruptured abdominal aortic aneurysm performed 13 years earlier. His medical history included myocardial infarction, atrial fibrillation, a left atrial appendage thrombus, and peripheral arterial occlusive disease. He was receiving long‐term antithrombotic therapy with aspirin, clopidogrel, and warfarin. Although the details were unclear, he had a documented history of contrast media allergy.

Over the preceding 3 months, the patient experienced five episodes of gastrointestinal bleeding, presenting with hematemesis, black vomitus, and dark‐red stools. He required hospitalization for each episode. Esophagogastroduodenoscopy (EGD) and colonoscopy were performed during every admission; however, no definitive source of bleeding was identified. Representative EGD images, along with laboratory data and transfusion history from each hospitalization, are provided in Document S1. Suspecting small intestinal bleeding, a capsule endoscopy was subsequently performed, but no abnormal findings were observed.

**FIGURE 1:**
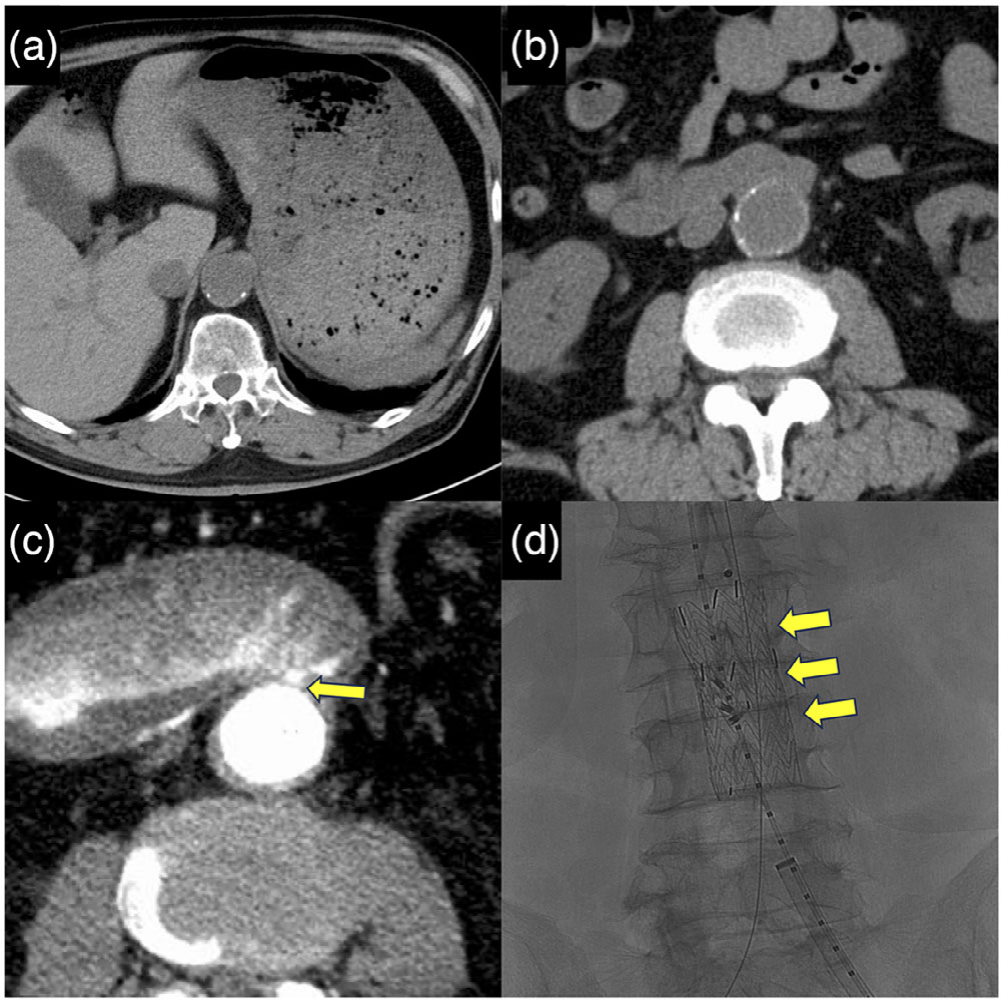
Computed tomography (CT) findings. (a) Non‐contrast CT revealing high‐attenuation material in the stomach, measuring approximately 40–50 Hounsfield units, suggestive of massive gastrointestinal bleeding. (b) Close anatomical proximity between the aorta and the horizontal portion of the duodenum; however, no aortic aneurysm or intraluminal air is detected. (c) Contrast‐enhanced CT demonstrating extravasation of contrast medium from the aorta into the horizontal portion of the duodenum, raising suspicion for secondary aortoduodenal fistula (arrow). (d) Emergency endovascular aortic repair (EVAR) resulted in hemostasis (arrow).

**FIGURE 2:**
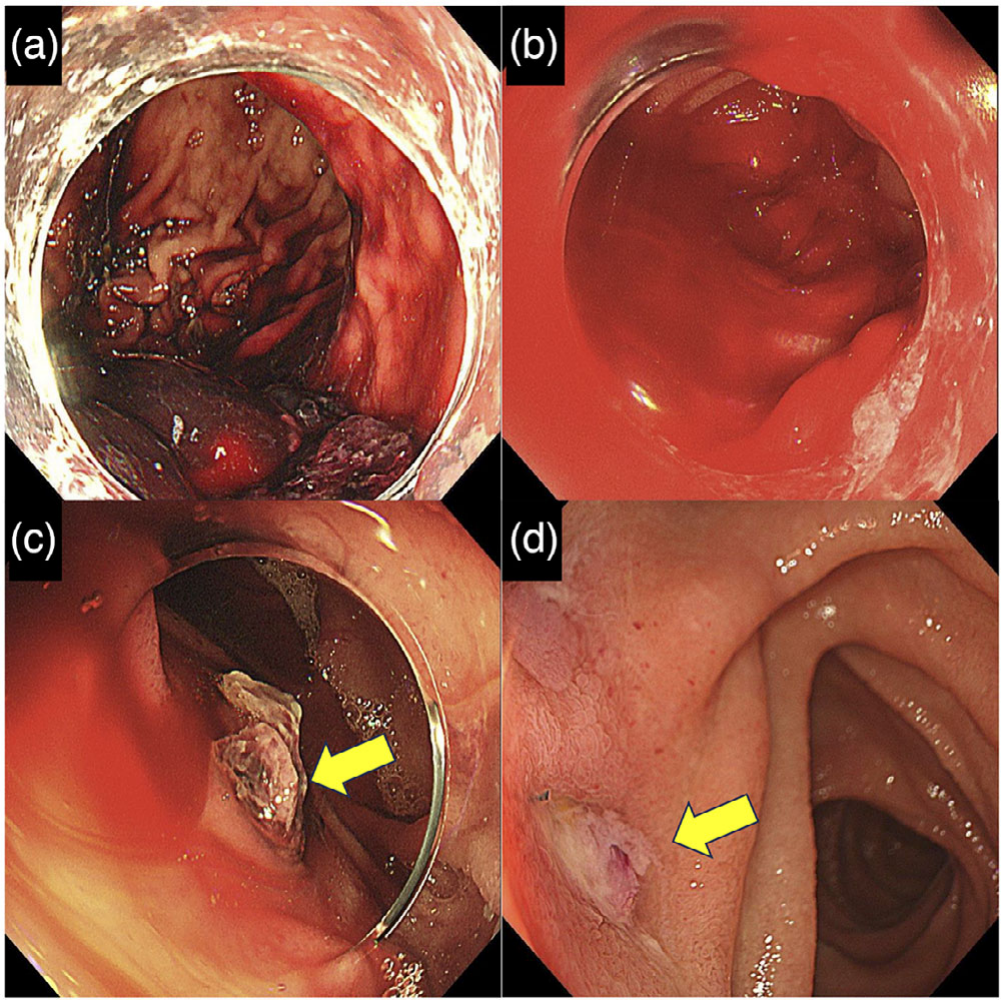
Endoscopic findings. (a) Initial endoscopy showing a large amount of blood residue in the stomach without identification of the bleeding source. (b) Emergency endoscopy during hypovolemic shock revealing massive blood in the stomach and duodenum, again without a clear bleeding source. (c) An exposed vessel identified in the horizontal portion of the duodenum (arrow). (d) Follow‐up endoscopy confirming the absence of active bleeding at the previously identified site (arrow).

Following another episode of hematemesis, the patient was brought to our hospital by ambulance. Upon arrival, his vital signs were stable, and physical examination revealed no significant abnormalities. Laboratory data demonstrated moderate anemia with a hemoglobin level of 8.0 g/dL. Blood urea nitrogen was 22.0 mg/dL, and creatinine was 1.0 mg/dL (Table 1). Due to his history of contrast media allergy, contrast‐enhanced CT could not be performed. Non‐contrast CT revealed high‐attenuation material within the stomach, measuring approximately 40–50 Hounsfield units, consistent with massive gastrointestinal bleeding (Figure 1a). The scan showed only anatomical proximity between the aorta and the horizontal portion of the duodenum, without evidence of an aortic aneurysm or intraluminal air (Figure 1b). All prior EGDs, as well as the current one, involved a standard upper endoscope (GIF‐H290T; Olympus). EGD showed a large volume of blood residue in the stomach but no definitive bleeding source (Figure 2a). The patient underwent a blood transfusion and was admitted for close monitoring.

On the day following admission, the patient developed hematemesis and fresh bloody stools. His blood pressure dropped to 75/51 mmHg, and his heart rate increased to 150 beats/min, indicating hypovolemic shock. Despite the initiation of an emergency blood transfusion, blood pressure remained unstable. A second EGD was therefore performed, which revealed large amounts of blood residue in the stomach and duodenum; however, no definitive source of bleeding could be identified (Figure 2b). As endoscopic hemostasis was not feasible, transfusion was continued, and the patient was monitored conservatively.

Approximately 4 h later, the patient again experienced hematemesis and hematochezia, leading to another episode of hypovolemic shock. After obtaining informed consent regarding the risk of contrast‐induced anaphylaxis, contrast‐enhanced CT was performed. The scan demonstrated extravasation of contrast medium from the aorta into the horizontal portion of the duodenum, raising suspicion for sADF (Figure 1c). Based on the findings of the contrast‐enhanced CT scan, a colonoscope (PCF‐H290T, Olympus) and a long colonoscope (PCF‐Q260L) were used to perform an upper endoscopy for detailed examination of the horizontal portion of the duodenum. This repeat EGD revealed an exposed vessel in the same area, confirming the diagnosis of sADF (Figure 2c). The vascular surgery team was consulted, and emergency endovascular aortic repair (EVAR) was performed, resulting in successful hemostasis (Figure 1d). The patient showed no further signs of bleeding postoperatively, and his condition gradually stabilized. Follow‐up endoscopy confirmed the absence of bleeding at the previously identified site (Figure 2d). He was discharged approximately 3 weeks after the EVAR.

## 3 Discussion

This case describes a 76‐year‐old man who developed sADF 13 years after aortic graft surgery. While this interval is relatively long, sADF can occur from within a few weeks to over 20 years after aortic graft surgery [6]. The clinical course—marked by inability to perform contrast‐enhanced CT and multiple negative endoscopic examinations lacking characteristic imaging findings—highlights the diagnostic challenges of sADF. This case offers important clinical insights: plain CT alone may fail to reveal specific signs of sADF; even during massive bleeding, endoscopic identification of the bleeding site can be difficult; and repeated episodes of mild gastrointestinal bleeding may be diagnostic clues (See Table 1).

**TABLE 1:** Laboratory data on admission.

Parameter	Value
White blood cells	8000/µL
Red blood cells	298 × 10⁴/µL
Hemoglobin	8.0 g/dL
Hematocrit	26.6%
Platelet count	21.9 × 10⁴/µL
Total protein	5.4 g/dL
Albumin	3.2 g/dL
Total bilirubin	0.4 mg/dL
Aspartate aminotransferase	19 U/L
Alanine aminotransferase	11 U/L
Alkaline phosphatase	48 U/L
Lactate dehydrogenase	234 U/L
Blood urea nitrogen	22.0 mg/dL
Creatinine	1.00 mg/dL
Sodium	141 mEq/L
Potassium	4.2 mEq/L
Chloride	108 mEq/L
C‐reactive protein	0.085 mg/dL
Prothrombin time—INR	1.71
Activated partial thromboplastin time	26.6 s

Diagnosing sADF is often challenging, as endoscopy and CT may not yield definitive findings. Although diagnosis often follows a major bleeding event, the bleeding source is frequently obscured during active hemorrhage. Up to 80% of sADF cases occur in the horizontal duodenum, making detailed inspection of this region essential [1, 6]. Therefore, careful observation of the horizontal portion of the duodenum is crucial when sADF is suspected. However, heavy bleeding often hampers visualization, as seen in our case, where multiple EGDs revealed only blood residue until an exposed vessel was finally detected [2, 5, 6]. CT can also be inconclusive. Contrast‐enhanced CT may reveal extravasation from the aorta into the duodenum, but this finding is not always present. In patients unable to undergo contrast‐enhanced imaging, indirect signs—such as aortic‐duodenal contact or intraluminal air—can be suggestive [2]. In our patient, plain CT failed to demonstrate these findings, delaying diagnosis. This case emphasizes the limitations of both endoscopy and CT, especially when contrast cannot be administered.

Alternative imaging modalities may be considered when contrast CT is contraindicated. Gadolinium‐enhanced MRI can evaluate bowel wall abnormalities but is suboptimal for acute GI bleeding [7]. Technetium‐99m–labeled red blood cell scans can detect bleeding rates of≥0.1 mL/min and may assist in identifying continuous bleeding; however, their sensitivity is limited in cases of intermittent bleeding, such as sADF [8]. Ultrasonography, while noninvasive and accessible, rarely visualizes aortoduodenal fistulas due to anatomical limitations [4]. Thus, despite their limitations, contrast‐enhanced CT and endoscopy remain the most reliable tools. When initial studies are inconclusive, repeated evaluations should be considered in patients with a history of aortic intervention and unexplained GI bleeding.

In this case, five hospitalizations for GI bleeding occurred over 3 months before sADF was diagnosed. This pattern is consistent with herald bleeding, which precedes fatal hemorrhage in over half of sADF cases [2, 6, 9]. Reports have described herald bleeding lasting over 130 days [4], highlighting the importance of recognizing this warning sign. In patients with aortic grafts, even intermittent minor bleeding episodes warrant a high index of suspicion.

Treatment options include open surgical repair (OSR) and EVAR. While OSR offers definitive closure and infection control, EVAR is preferred for patients with hemodynamic instability or poor surgical candidacy due to its minimally invasive nature. EVAR is effective as an emergency intervention but carries risks of infection and fistula persistence, necessitating close follow‐up [6, 10]. In our case, EVAR achieved prompt hemostasis without complications.

This case illustrates that even without classic imaging findings, a history of aortic grafting combined with recurrent unexplained GI bleeding should prompt early consideration of sADF.

## Ethics Statement

Not applicable.

## Conflicts of Interest

The authors declare no conflicts of interest.

## Supporting information




**DOCUMENT S1** Summary of the patient's five prior hospitalizations, including representative endoscopic images, laboratory data, and transfusion history.

## References

[1] S. J. F. Saers and M. R. M. Scheltinga , “Primary Aortoenteric Fistula,” British Journal of Surgery 92, no. 2 (2005): 143–152. 15685700 10.1002/bjs.4928

[2] C. Ichita , A. Sasaki , C. Sumida , et al., “Clinical and Endoscopic Features of Aorto‐duodenal Fistula Resulting in Its Definitive Diagnosis: An Observational Study,” BMC Gastroenterology [Electronic Resource] 21, no. 1 (2021): 45. 33526013 10.1186/s12876-021-01616-9 PMC7851914

[3] R. Voorhoeve , F. L. Moll , J. A. de Letter , T. J. Bast , J. P. Wester , and P. H. Slee , “Primary Aortoenteric Fistula: Report of Eight New Cases and Review of the Literature,” Annals of Vascular Surgery 10, no. 1 (1996): 40–48. 8688296 10.1007/BF02002340

[4] C. L. Deijen , Y. M. Smulders , H. M. E. Coveliers , W. Wisselink , J. A. Rauwerda , and A. W. J. Hoksbergen , “The Importance of Early Diagnosis and Treatment of Patients With Aortoenteric Fistulas Presenting With Herald Bleeds,” Annals of Vascular Surgery 36 (2016): 28–34. 27423720 10.1016/j.avsg.2016.03.028

[5] M. Bala , J. Sosna , L. Appelbaum , E. Israeli , and A.‐I. Rivkind , “Enigma of Primary Aortoduodenal Fistula,” World Journal of Gastroenterology 15, no. 25 (2009): 3191–3193. 19575502 10.3748/wjg.15.3191 PMC2705745

[6] N. Matsuura , K. Fujitani , R. Nakatsuka , et al., “Secondary Aortoduodenal Fistula: Report of 3 Cases,” Japanese Journal of Gastroenterological Surgery 51, no. 6 (2018): 406–414.

[7] J. Rimola , J. Torres , S. Kumar , S. A. Taylor , and T. Kucharzik , “Recent Advances in Clinical Practice: Advances in Cross‐sectional Imaging in Inflammatory Bowel Disease,” Gut 71, no. 12 (2022): 2587–2597. 35927032 10.1136/gutjnl-2021-326562 PMC9664122

[8] N. Sengupta , D. M. Kastenberg , D. H. Bruining , et al., “The Role of Imaging for Gastrointestinal Bleeding: Consensus Recommendations From the American College of Gastroenterology and Society of Abdominal Radiology,” American Journal of Gastroenterology 119, no. 3 (2024): 438–449. 38857483 10.14309/ajg.0000000000002631

[9] M. Batt , E. Jean‐Baptiste , S. O'Connor , et al., “Early and Late Results of Contemporary Management of 37 Secondary Aortoenteric Fistulae,” European Journal of Vascular and Endovascular Surgery 41, no. 6 (2011): 748–757. 21414817 10.1016/j.ejvs.2011.02.020

[10] A. Bartley , S. T. Scali , S. Patterson , et al., “Improved Perioperative Mortality After Secondary Aortoenteric Fistula Repair and Lessons Learned From a 20‐year Experience,” Journal of Vascular Surgery 75, no. 1 (2022): 287–295. 34303801 10.1016/j.jvs.2021.07.107

