# Supplementary material for: Secondary Aortoduodenal Fistula Diagnosed after Repeated Gastrointestinal Bleeding Episodes in a Patient With Prior Aortic Graft Surgery
Source: DEN Open. 2025 Aug 6;6(1):e70181. doi: 10.1002/deo2.70181 (PMC12328087; doi:10.1002/deo2.70181)

## Summary of Prior Hospitalizations

This supplementary material provides a summary of the patient’s five prior hospitalizations, including endoscopic images, laboratory data, and transfusion history. All hospitalizations occurred at our institution prior to the definitive diagnosis of secondary aortoduodenal fistula (sADF).

### Data from the five hospitalizations prior to diagnosis


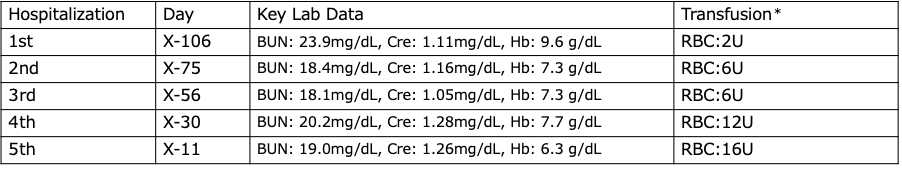


### Endoscopic image (first hospitalization)


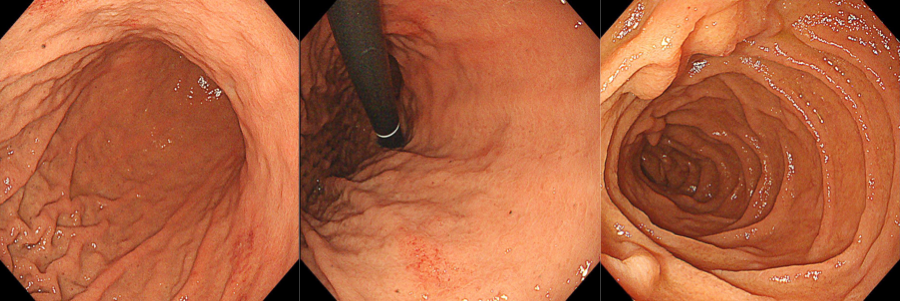


### Endoscopic image (second hospitalization)


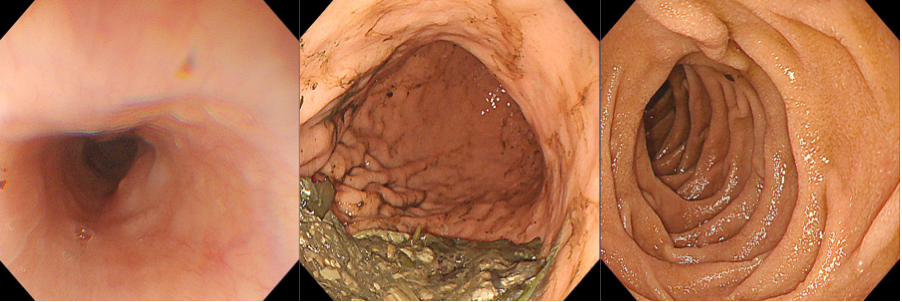


### Endoscopic image (third hospitalization)


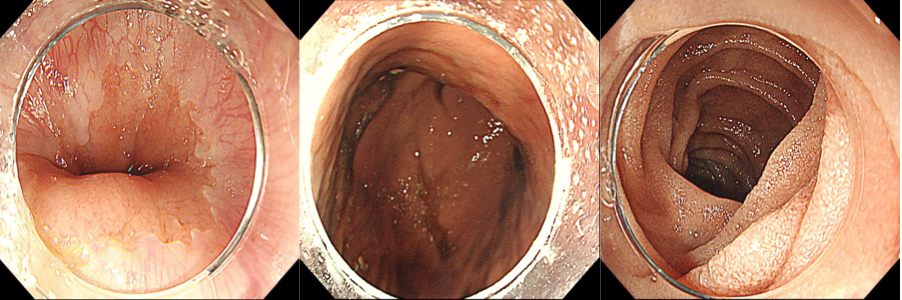


### Endoscopic image (fourth hospitalization)


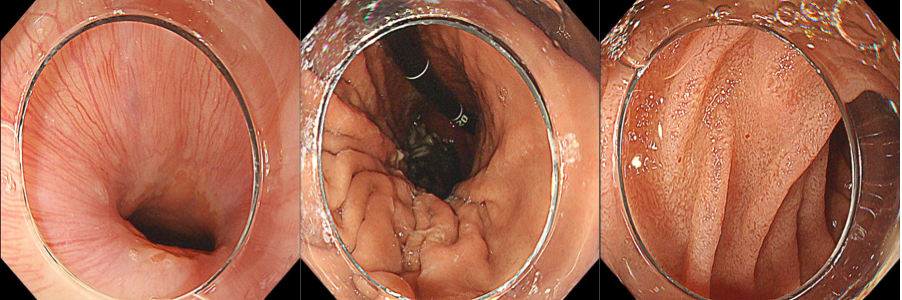


### Endoscopic image (fifth hospitalization)


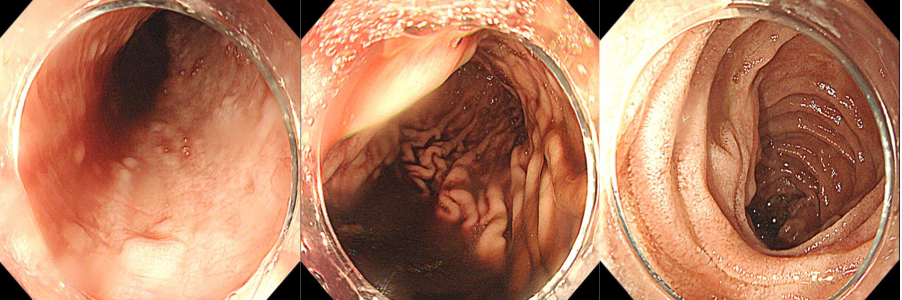

Supplement: Supplementary file 1 — DOCUMENT S1 Summary of the patient's five prior hospitalizations, including representative endoscopic images, laboratory data, and transfusion history. [file DEO2-6-e70181-s001.docx]
